# Central hypoventilation with hypercapnia: the silent face of ventilatory control

**DOI:** 10.36416/1806-3756/e20260320

**Published:** 2026-09-14

**Authors:** Izabella M Brito Marques, Mariana Lima Lafetá, Eloara V M Ferreira

**Affiliations:** 1. Divisão de Pneumologia, Escola Paulista de Medicina, Universidade Federal de São Paulo - UNIFESP - São Paulo (SP) Brasil.

## INTRODUCTION

Central hypoventilation arises from impaired neural regulation of ventilation, resulting in inadequate alveolar ventilation to meet metabolic requirements and subsequent carbon dioxide retention. This condition typically manifests without significant parenchymal lung disease, neuromuscular disorders, or mechanical abnormalities of the respiratory system that could otherwise account for hypoventilation.^1^ ^,^ ^2^ Cardiopulmonary exercise testing (CPET) serves as a valuable diagnostic tool for patients with unexplained dyspnea or hypoxemia, enabling a comprehensive assessment of ventilatory, cardiovascular, and metabolic responses during exercise.^3^

## OVERVIEW

A 51-year-old non-obese woman (BMI = 23.3 kg/m²) with hypertension and diabetes, treated with losartan, metoprolol, and metformin, presented with a history of two respiratory infections associated with hypoxemia requiring oxygen therapy during hospitalization. In the outpatient setting, she exhibited mild dyspnea and exercise-induced hypoxemia during a two-minute step test (SpO_2_, 91% at rest and 78% at peak exercise). Spirometry revealed reduced FVC (68% of the predicted value) and FEV_1_ (1.99 L; 75% predicted), with a normal FEV_1_/FVC ratio (0.89). Blood workup, chest CT angiography, and microbubble echocardiography were unremarkable. Two years later, CPET demonstrated low VO_2_peak with a normal anaerobic threshold and oxygen pulse. Peak minute ventilation was low with normal Vt and no change in respiratory rate, accompanied by a reduced Ve/VCO_2_ slope (Ve/VCO_2_ = 12) and a progressive increase in end-tidal carbon dioxide pressure (PetCO_2_) from 44 mmHg at rest to 69 mmHg at peak exercise. Exercise-induced hypoxemia was also observed (SpO_2_, 95% at rest and 68% at peak exercise), suggesting a ventilatory control disorder (Figure 1A). 

**Figure 1:**
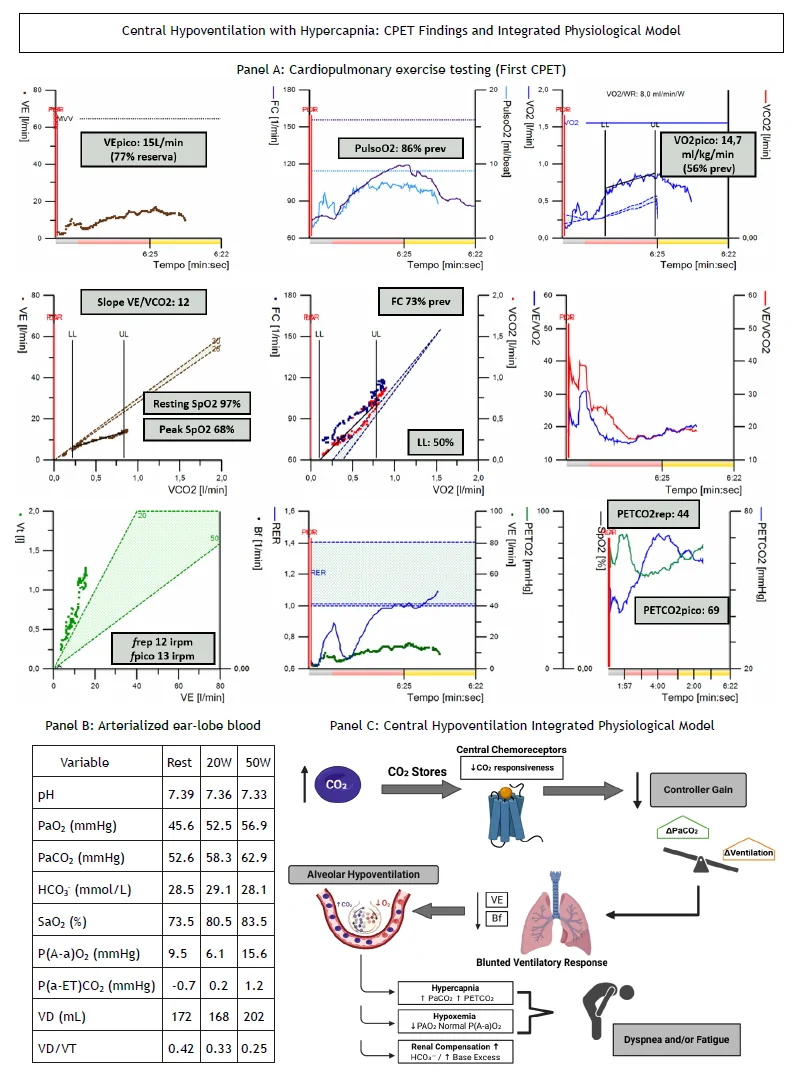
Integrated physiological assessment of central hypoventilation with hypercapnia. In A, cardiopulmonary exercise testing demonstrating an inadequate ventilatory response during exercise, characterized by reduced peak minute ventilation (Ve) related to low breathing frequency (Bf), ventilatory and gas exchange impairment, low Ve/VCO_2_ slope, progressive increase in PetCO_2_ during exercise reaching elevated levels at peak exercise, and hypoxemia. In B, cardiopulmonary exercise testing (CPET) with arterialized earlobe blood gas analysis (CPET level 2) was performed after six months of BiPAP therapy during sleep demonstrating chronic respiratory acidosis, with normal pH and elevated bicarbonate and base excess, and hypoxemia (PaO_2_) with normal alveolar-arterial oxygen gradient. Despite hypercapnia, the arterial-end-expiratory CO_2_ pressure gradient was normal, indicating preserved pulmonary gas exchange efficiency. The dead space to tidal volume fraction (Vd/Vt) was increased at rest and during exercise, consistent with a low increment in Vt. In C, the pathophysiological model illustrates how reduced central chemoreceptor responsiveness decreases controller gain (ΔPaCO_2_/ΔVe), leading to a blunted ventilatory response with reduced Ve and Bf. The resulting alveolar hypoventilation promotes CO_2_ retention, hypercapnia, hypoxemia with a normal alveolar-arterial oxygen gradient, followed by renal compensation and the development of dyspnea and/or fatigue.

Polysomnography identified both central (61 events) and obstructive (353 events) sleep apnea, raising suspicion for a central hypoventilation syndrome. Blood gas analysis revealed PaCO_2_ of 62 mmHg and bicarbonate of 34 mEq/L. Following initiation of BiPAP therapy, repeat CPET with arterialized earlobe blood gas sampling showed persistent hypoventilation, chronic respiratory acidosis, and hypoxemia, with a normal alveolar-arterial oxygen gradient (Figure 1B). 

In patients with unexplained dyspnea and hypoxemia, disorders of ventilatory control should be considered. Hypoventilation syndromes comprise a heterogeneous group of disorders characterized by hypercapnia (PaCO_2_ ≥ 45 mmHg at sea level) due to impaired respiratory homeostasis. The differential diagnosis for central hypoventilation includes congenital central alveolar hypoventilation (Ondine syndrome), idiopathic hypoventilation, and medication-induced causes, such as chronic opioid or benzodiazepine use. Additional considerations include obesity hypoventilation syndrome, neuromuscular dystrophies, post-polio syndrome, and restriction of thoracic or abdominal expansion. 

In the present case, the progressive increase in PetCO_2_ with a normal Vt during exercise was consistent with alveolar hypoventilation secondary to impaired ventilatory control. Pulmonary ventilation is regulated by the respiratory center in the brainstem, which integrates input from central and peripheral chemoreceptors. Central chemoreceptors primarily respond to changes in PaCO_2_, which is a major determinant of ventilatory drive.^1^ ^,^ ^2^ When this stimulus is diminished, alveolar ventilation becomes inadequate to eliminate muscle-derived CO_2_, resulting in increased PetCO_2_ in expired air and, with worsening hypoventilation, hypercapnia.^3^ ^,^ ^4^

Chronic hypercapnia also induces renal compensatory mechanisms, including increased bicarbonate reabsorption and partial metabolic compensation for respiratory acidosis (Figure 1C).^4^

From a pathophysiological perspective, the alveolar gas equation, as follows:

PAO2=[Pb–PH2O]×FiO2–PaCO2/RER

where Pb is barometric pressure, PH_2_O is water vapor pressure, FiO_2_ is the fraction of inspired oxygen, and RER is the respiratory exchange ratio, illustrates how these parameters contribute to hypoxemia. Hypoxemia may result from reduced oxygen uptake in the lungs due to environmental factors such as high altitude or low inspired oxygen fraction, as well as from an altered ventilatory response relative to CO_2_ output. Reduced alveolar ventilation decreases the delivery (minute ventilation) of fresh air to the alveoli, lowering PAO_2_, reducing oxygen diffusion into the pulmonary capillary blood, and causing a progressive decline in PaO_2_. In this context, the alveolar-arterial oxygen gradient typically remains normal or only mildly elevated, a hallmark of alveolar hypoventilation. 

## CLINICAL MESSAGE

Disorders of ventilatory control can result in inadequate alveolar ventilation relative to metabolic CO_2_ production, leading to hypercapnia and hypoxemia even in the absence of structural lung disease. CPET with blood gas analysis may provide critical physiological insights by revealing an inadequate ventilatory response to metabolic demand during exercise.^3^
